# 基于低共熔溶剂的多孔有机框架材料合成及其在固相萃取中的应用

**DOI:** 10.3724/SP.J.1123.2023.08025

**Published:** 2023-10-08

**Authors:** Wenqian JIANG, Yumei CHEN, Wentao BI

**Affiliations:** 南京师范大学化学与材料科学学院, 江苏 南京 210023; School of Chemistry and Materials Science, Nanjing Normal University, Nanjing 210023, China

**Keywords:** 金属有机框架, 共价有机框架, 低共熔溶剂, 固相萃取, 综述, metal-organic frameworks (MOFs), covalent organic frameworks (COFs), deep eutectic solvent (DES), solid-phase extraction (SPE), review

## Abstract

本文综述了低共熔溶剂(DES)在多孔有机框架材料(POFs)中金属有机框架(MOFs)和共价有机框架(COFs)合成方面的应用,以及这些新型材料在固相萃取领域的潜在应用。DES作为环保绿色溶剂,不仅用于MOFs和COFs的制备,还在特定情况下作为结构导向剂,对框架的结构和性能产生重要影响。通过合适的DES配方,研究人员能够调控MOFs和COFs的晶体结构、孔径和表面性质,从而获得性能卓越的材料。MOFs和COFs因其具有较大的比表面积和丰富的活性位点,在固相萃取中展现出卓越的吸附能力和选择性,能够有效地从复杂样品中富集目标分析物。研究证明,基于DES的MOFs和COFs在环境分析、食品检测和生物样品分析等领域具有广泛的应用潜力。尽管基于DES的MOFs和COFs在固相萃取领域仍处于初级阶段,但其高效富集和高选择性等特性为实际应用提供了良好前景。未来的研究应继续深入探索基于DES的合成方法,以制备更多性能卓越的MOFs和COFs,并深入研究其在各个领域的应用潜力。这些努力有望将这些新型材料应用于商业化的固相萃取方法中,为分析化学领域带来新的发展机遇。

## Abstract (en)

This paper reviews the application of deep eutectic solvents (DESs) in the synthesis of metal-organic frameworks (MOFs) and covalent organic frameworks (COFs) as well as their prospects in the field of solid-phase extraction (SPE). Porous organic frameworks (POFs) have unique properties such as a large specific surface area, high porosity, and easy modification. Thus, these materials are widely applied in the fields of catalysis, adsorption, drug delivery, gas storage, and separation. POFs include MOFs, COFs, conjugated microporous polymers (CMPs), porous aromatic frameworks (PAFs), and covalent triazine frameworks (CTFs). MOFs are constructed from metal ions/clusters and organic ligands through coordination bonds and can be extended in two or three dimensions by repeated coordination with potential voids. COFs are formed from two monomers containing light elements (such as carbon, hydrogen, oxygen, nitrogen, boron, and other elements) via coordination bonds and have large two- or three-dimensional structures. However, conventional POF synthesis methods generally suffer from disadvantages such as long synthesis times, high temperature and pressure requirements, and the use of toxic and hazardous reaction solvents. DES consists of a hydrogen bond acceptor (HBA) and a hydrogen bond donor (HBD) bound by hydrogen-bonding interactions. It is a promising green solvent for material synthesis owing to its low vapor pressure, high stability, and ease of preparation. DES can be used to prepare MOFs and COFs and, in specific cases, acts as a structure-directing agent, which has an important impact on the structure and properties of the resulting frameworks. Using appropriate DES formulations, researchers can modulate the crystal structures, pore sizes, and surface properties of MOFs and COFs, resulting in materials with excellent characteristics. SPE is an analytical technique in which a sample solution is added to an SPE column; the sample solution is forced through the stationary phase, and the target compounds are collected for analysis by elution with an organic solvent. Therefore, suitable stationary-phase materials are critical for SPE. Owing to their large specific surface areas and abundant active sites, MOFs and COFs exhibit outstanding adsorption capacity and selectivity in SPE and can effectively enrich target analytes from complex samples. DES-based MOFs and COFs have shown potential use in a wide range of applications, such as in environmental analysis, food testing, and biological sample analysis. Although DES-based MOFs and COFs for SPE are still in the early stages of development, their properties such as efficient enrichment and high selectivity offer good prospects for practical applications. Future research should continue to explore DES-based synthesis methods in depth to prepare other MOFs and COFs with the desired properties and investigate their potential applications in various fields. These efforts are expected to apply these novel materials in commercialized solid-phase extraction methods, bringing new development opportunities in the field of analytical chemistry.

近些年来,多孔有机框架材料(POFs)由于具有极高的比表面积、高孔隙率和可设计性等优点,已经从传统多孔材料中脱颖而出^[1]^。POFs是指一类含有有机单元的多孔框架材料,其中包括金属有机框架材料(MOFs)、共价有机框架材料(COFs)、共轭微孔聚合物材料(CMPs)、多孔芳香框架材料(PAFs)以及共价三嗪框架材料(CTFs)^[2]^。其中,MOFs由金属离子/簇和有机配体通过配位键构建^[3]^。金属离子簇和有机配体的多功能性以及它们在各种方向上的广泛连接方式,决定了MOFs的结构多样性和丰富的物理、化学性质。与MOFs类似,COFs也是一类具有周期性有序结构的多孔2D或3D有机材料。不同之处在于,COFs由含有轻质元素(如C、H、O、N、B等)的单体通过共价键构建^[4]^。CMPs是具有*π*共轭骨架的聚合物,由孔径小于或等于2 nm的纯共价有机材料构建而成^[5,6]^。PAFs由碳-碳键连接的芳香族构建单元组成,各种功能既可来自其结构单元的内在化学性质,也可通过既定反应对芳香基团进行修饰来实现^[7]^。CTFs通常通过腈类芳香族结构单元的环化反应构建而成,除芳香基团外不存在弱键^[8,9]^。然而,传统的POFs合成方法一般合成时间较长,对温度和压强要求较高,或者使用了有毒有害的反应溶剂,存在一定的局限性^[10⇓-12]^。

低共熔溶剂(DES)是一种新型的离子液体(IL)类似物,被认为是除传统IL外的另一类新型环保液体材料。如图1所示,DES由氢键受体(HBA)与氢键供体(HBD)通过氢键作用力结合^[13]^,它既不需要通过有机溶剂进一步纯化,也不需要后续处理,合成后即可使用,并且原子使用率为100%,因此是真正的绿色溶剂。与IL相似,DES具有蒸气压低、热稳定性高、不易燃和制备简便等特性。此外,DES还具有价格低廉、无毒害、可生物降解等优点。这些优良的物理化学特性使得DES成为合成制备POFs的良好溶剂。然而,遗憾的是,目前仅有基于DES合成MOFs和COFs的相关研究。大部分MOFs和COFs具有丰富的活性位点、良好的可重复使用性和稳定性等特点,因此在固相萃取(solid-phase extraction, SPE)领域引起了广泛的关注^[14]^。本文总结了基于绿色溶剂DES合成MOFs和COFs的方法,并综述了近5年来MOFs及COFs在固相萃取中的应用。

**图 1:**
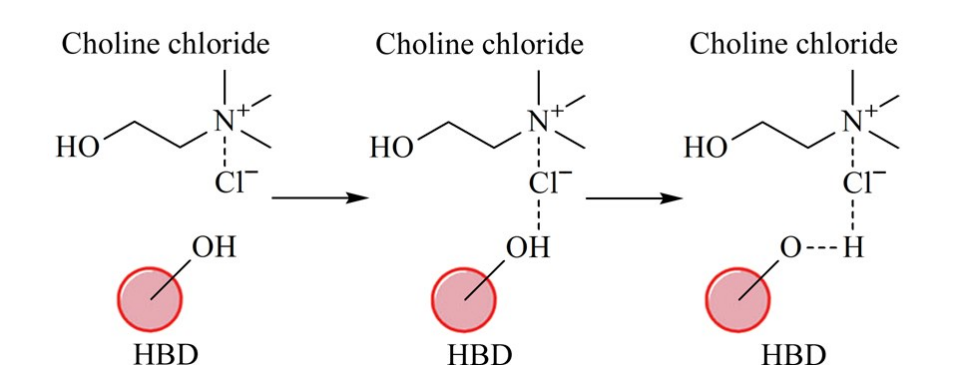
低共熔溶剂中氢键受体氯化胆碱与氢键供体的相互作用^[13]^

## 1 基于DES的有机框架合成

与传统MOF、COF合成途径相比,在DES中合成MOF、COF的反应条件更加简便、环保。并且,DES在MOF和COF形成过程中可以扮演不同的角色,既可以作为溶剂,也可以作为结构导向剂(structure-directing agent, SDA)^[15]^,如图2所示。DES的成分或分解产物之一可能会存在于MOF或COF的结构中,可能作为一个单体与另一个单体组合,或者存在于其孔中,从而对晶体结构产生一定的影响。这是传统的溶剂热法无法实现的。

**图 2:**
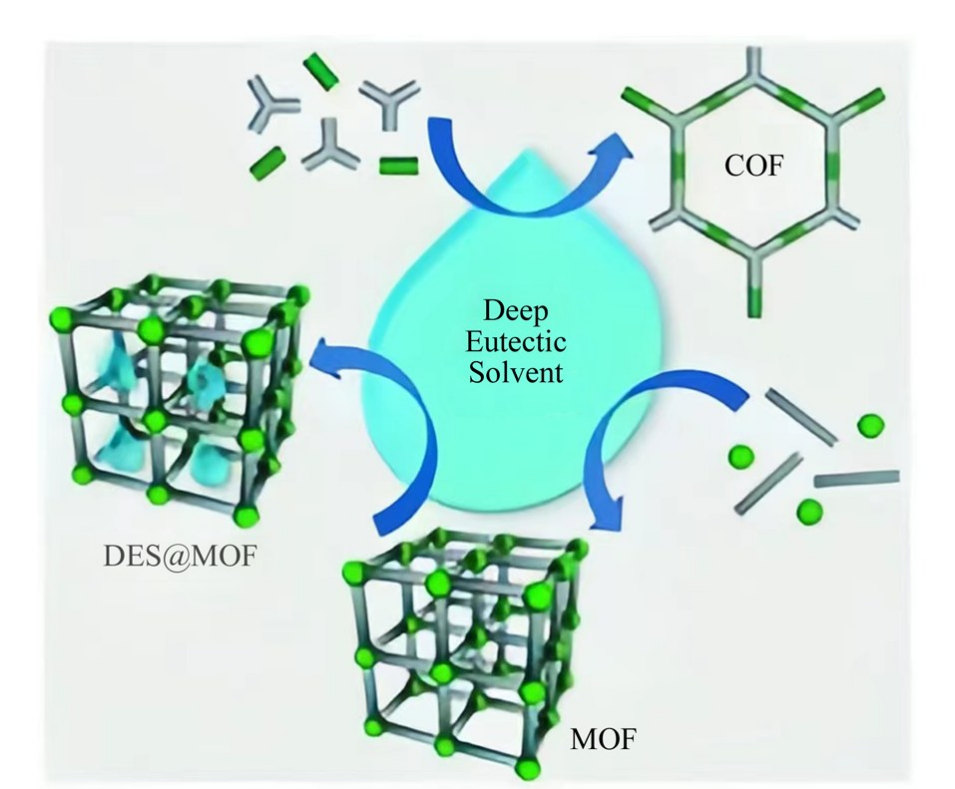
基于DES合成MOFs和COFs的示意图^[15]^

### 1.1 基于DES合成MOF

#### 1.1.1 DES在MOF合成中作溶剂

使用DES作为反应溶剂合成MOF具有多重优势,包括降低有机试剂的使用量,提升实验的安全性,并降低对反应环境中温度和压力的要求。一些研究者已经在尿素基的DES中成功合成MOF,以实现这些目标。例如Maia等^[16]^使用由氯化胆碱(choline chloride, ChCl)和尿素(urea)组成的DES作为溶剂,将Cu(NO_3_)_2_·3H_2_O和均苯三甲酸(H_3_BTC)混合后,在超声处理和温和(无需高温高压)反应条件下,成功合成了HKUST-1。所获得的材料不仅具备良好的结构性能,还呈现出独特的玫瑰状形态。Teixeira等^[17]^采用相同的DES作为溶剂,成功合成了Mg-MOF-74。他们在更加环保的条件下进行了CO_2_吸附实验,取得了良好的结果,建立了取代传统有机溶剂的绿色、安全的合成策略。Patil等^[18]^也采用类似的DES溶剂,将对苯二甲酸(phthalic acid, PTA)和Cu(NO_3_)_2_·3H_2_O混合,在较为温和的条件下成功合成了浅蓝色Cu-BDC材料。与传统的合成方法相比,这种合成策略不仅在能量消耗上节省了97%,还能够减少反应过程中的环境影响。

尿素基DES通常较为黏稠。为了促进合成过程中的物质交换,通常引入适量的去离子水以降低其黏稠度。Hu等^[19]^将去离子水与尿素基DES混合(去离子水质量分数为15%)作为溶剂,并在60 ℃下进行搅拌以溶解十二烷基硫酸钠(SDS)、Zn(NO_3_)_2_·6H_2_O和Co(NO_3_)_2_·6H_2_O。随后,添加了一定量的二甲基咪唑酯(2-methylimidazole, 2-Mim),并在60 ℃条件下搅拌1 h,最后通过离心、洗涤、干燥后成功合成Co/ZIF-8。研究结果显示,与传统材料相比,所得的Co/ZIF-8具有更大的比表面积、孔体积以及优良的热稳定性。此外,该材料还呈现出微-中-大孔的分层多孔结构。

此外,Hu等^[20]^采用由2-Mim和四丁基溴化铵(tetra-*n*-butylammonium bromide, TBAB)组成的咪唑酯基DES作为溶剂。在这个实验中,他们混合了Zn(NO_3_)_2_·6H_2_O、2-Mim和TBAB,然后在40 ℃下搅拌反应30 min,形成均匀的无色溶液。在加入去离子水后,溶液迅速变成乳白色。最后通过离心、洗涤、干燥,成功合成出ZIF-8。这种合成过程不含有机溶剂,在室温下进行且反应时间短。这是由于季铵盐与咪唑配体之间的氢键相互作用会形成DES,促进咪唑配体的去质子化,进而促进咪唑配体与Zn^2+^的配位,从而加速了ZIF-8的成核和生长。

#### 1.1.2 DES在合成MOF中作结构导向剂

Maia等^[16]^在尿素基DES中成功合成了呈现玫瑰花状形态的HKUST-1。与传统合成方法相比,这种新型合成方法制备的HKUST-1在形貌上存在显著差异。此外,研究团队还观察到另一种MOF结构Cu_2_(BTC)Cl的形成。这是因为在MOF的合成过程中,DES充当了结构导向剂的角色,诱导出两种不同的MOF结构。这种DES引导作用导致了不同形态的MOF产生转变。Teixeira等^[17]^基于含尿素和乙烯-脲(ethylene urea, e-urea)的DES,成功合成了具有多种中间晶相的Mg-MOF-74。通过X射线衍射分析,研究人员发现DES组分中的Cl^-^和e-urea衍生物阴离子与Mg(Ⅱ)阳离子配位,与羧酸连接剂竞争配位。这表明DES在MOF的合成中充当了结构导向剂的角色,影响了MOF的形态。因此,Mg-MOF-74的形态与传统的棒状结构不同,而是呈现出垂直于主要生长轴的薄片状结构。这种非常规形态的Mg-MOF-74材料具有更大的比表面积和微孔隙度,从而使其对CO_2_的吸附能力增强。

Teixeira等^[21]^同样以ChCl和e-urea组成的DES作为溶剂,掺杂一定量的Ca(NO_3_)_2_·4H_2_O和一系列二羧酸配体,成功合成出一系列的不同结构形态的Ca-MOF。这些MOF的结构中包含通道,其中DES在不同MOF中扮演不同的角色,有时作为桥接配体,有时作为末端配体。这种差异是由DES在MOF合成中的结构引导作用导致的。值得注意的是,Ca(dobdcH_2_)(e-urea)_2_晶体在空气中暴露后迅速失去了透明度和光泽,转变为另一个相Ca_2_(dobdcH_2_)_2_(e-urea)_3_(H_2_O) (e-urea),这是由于Ca(Ⅱ)阳离子配位球中的一个e-urea分子被环境中的水分子取代,然后被释放到MOF的孔隙中。

Hu等^[20]^采用DES成功制备了ZIF-8,在这个方法中,季铵盐作为模板,促使ZIF-8材料具有卓越的比表面积和孔隙体积,并实现微孔和介孔的共存。相比之下,传统溶液法制备的ZIF-8只包含微孔,这使其在CO_2_吸收方面表现出更高的效能。Yin等^[22]^采用由铵盐和乙酰胺构成的DES,其水的质量分数为20%,混合一定量的ZrCl_4_和PTA后,将混合物放入圆底烧瓶中。然后,在100 ℃条件下磁力搅拌(800 r/min)5 h,最终通过离心、洗涤和干燥过程合成出非晶态UiO-66材料。在所合成的非晶态UiO-66样品中,具有缺陷无序结构的样品表现出最高的含量,对丙烯磷表现出显著的亲和力,其晶体吸附量是晶态样品的3倍。

### 1.2 基于DES合成COF

#### 1.2.1 DES在COF合成中作溶剂

在2020年,Qiu等^[23]^首次在DESs中成功合成了多个2D和3D COFs。以氯化胆碱-甘油(choline chloride-glycerol, ChCl-Gly)、氯化胆碱-乙二醇(choline chloride-ethylene glycol, ChCl-EG)和氯化胆碱-尿素(choline chloride-urea, ChCl-Urea)等DESs作为溶剂,在室温下将1,3,5-三甲酰基间三苯酚(Tp)分别与对苯二胺(Pa)、肼(Ha)、4,4'-偶氮二苯胺(Azo)和4,4'-二氨基-2,2'-联吡啶(Bpy)进行搅拌反应,成功合成了COFs,并将其命名为TpPa、TpHa、TpAzo和TpBpy。其中,与传统方法合成的TpPa相比,TpPa-Gly的合成时间从72 h大幅缩短至2 h,并且获得了高收率(90%)的结晶固体。由于COF单体完全暴露于DES中,实验获得了高结晶度、高稳定性的TpPa-Gly。在沸水、3 mol/L NaOH和3 mol/L HCl水溶液中保存3天,TpPa-Gly都能保持良好的晶体结构。此外,TpPa-Gly、ChCl-EG和ChCl-Urea的比表面积分别为747、805和977 m^2^/g,均远高于传统方法合成的TpPa(535 m^2^/g),并且可以通过调整DES中的氢键受体和氢键供体,调节COF的结构与性质。与2D COF相比,3D COF具有更高的比表面积、更多的活性位点。然而,3D COF的结构复杂且网络拓扑有限,已发表的3D COF仍比较少。受上述方法的启发,成功在ChCl-Gly中合成高结晶度的3D-COF-1和3D-COF-HNU10。HNU10是一种新型偶氮基3D COF,并伴有均匀的纳米棒结构。当尝试使用传统的溶剂热法和不同类型的有机溶剂合成3D-COF-HNU10时,只能得到无序聚合物,无法获得该COF。因此,可以认为这一策略推动了3D COF材料合成的发展。2022年,Xiao等^[24]^以ChCl和二水合草酸(oxalic acid dihydrate, OA)组成的DES为溶剂,以Tp和Pa为COF单体,在室温下搅拌合成了蜂窝状的TpPa。X射线光电子能谱显示只存在对应于C、N和O元素的3个主峰,并不存在与Cl元素相关的峰,这表明作为反应介质的DES在合成的TpPa中没有额外的残留。

2021年,Chen等^[25]^以四丁基溴化铵(Bu_4_NBr)和咪唑(imidazole, Im)组成的DES作为溶剂,以2,4,6-三(5-甲酰基-2-吡啶氧基)-1,3,5-三嗪(TFPT)和肼(Ha)为COF单体,在90 ℃下的开放容器中合成了含C=N键的TFPT-Azine-COF。随后,将TFPT-Azine-COF浸渍在CH_2_Cl_2_的Pd(OAc)_2_溶液中,制备了Pd/TFPT-Azine-COF非均相催化剂。热重分析表明,TFPT-Azine-COF与Pd/TFPT-Azine-COF的热分解温度均在300 ℃左右,因此具有优异的热稳定性。通过透射电子显微镜(TEM)可以发现,Pd均匀地分布在TFPT-Azine-COF载体之中,表明TFPT-Azine-COF结构具有良好的有序性和周期性。

Gao等^[26]^将由ChCl和六氟异丙醇(HFIP)组成的DES和1,3,5-三(4-氨基苯基)苯(TAPB)、2,5-二羟基对苯二甲醛(DHA)两种COF单体混合于玻璃安瓿管中,先超声处理10 min,再在液氮中进行3次冷冻-泵-融化循环,最后在80 ℃下保持48 h得到COF-DES。热重分析表明,COF-DES在400 ℃以下稳定重量损失很小(9.97%),重量的损失归因于COF-DES孔隙中残留溶剂的损失。温度为400~800 ℃时,发生急剧的重量损失(29.07%),这归因于COF框架的部分碳化与分解,表明COF-DES具有良好的热稳定性。

#### 1.2.2 DES在COF合成中的其他作用

在高温、有毒有机溶剂、酸性催化剂、长反应时间等非绿色的恶劣条件下合成的COF会导致酶失活^[27]^。在COF中原位嵌入酶比较困难,因此需要开发新型绿色方法来合成COF用于包埋酶。Talekar等^[28]^以ChCl和尿素组成的DES为溶剂和催化剂,促进COF单体在酶表面形成“盔甲状”的COF框架,从而实现将酶包埋于COF之中,并且不用考虑COF孔径与酶的大小相对关系。DES是一种新型绿色溶剂,本身即可用于多种酶反应。在ChCl-Urea DES中合成的包埋了葡萄糖氧化酶(GOx)的COF复合材料GOx@COF保持了COF的高比表面积和孔隙率,使底物与产物之间能够充分传质。综上,表1详细总结了基于DES合成MOF和COF的方法。

**表 1:** 基于DES合成MOFs和COFs及DES在合成中的作用

Ref.	DESs			Synthetic MOF/COF monomer		MOF/COF	Function of DES
	HBA	HBD		Ligand	Metal-salt		
[16]	ChCl	urea		H_3_BTC	Cu(NO_3_)_2_·3H_2_O	HKUST-1	solvent, SDA
[17]	ChCl	urea		DHTA	Mg(NO_3_)_2_·6H_2_O	Mg-MOF-74	solvent
	ChCl	e-urea					solvent, SDA
[18]	ChCl	urea		PTA	Cu(NO_3_)_2_·3H_2_O	Cu-BDC	solvent
[19]	ChCl	urea		SDS	Zn(NO_3_)_2_·6H_2_O,	Co/ZIF-8	solvent
					Co(NO_3_)_2_·6H_2_O		
[20]	2-Mim	TBAB		2-Mim	Zn(NO_3_)_2_·6H_2_O	ZIF-8	solvent, SDA
[21]	ChCl	e-urea		dicarboxylic acid	Ca(NO_3_)_2_·4H_2_O	Ca-MOF	solvent, SDA
[22]	ammonium salts	acetamide		PTA	ZrCl_4_	Amorphous UiO-66	solvent, SDA
[23]	ChCl	Gly/EG/urea		Tp	Pa	TpPa	solvent
					Ha	TpHa	
					Azo	TpAzo	
					Bpy	TpBpy	
				TFPM	Pa	3D-COF-1	
					Azo	3D-COF-HNU10	
[24]	Bu_4_NBr	Im		TFPT	Ha	TFPT-Azine-COF	solvent
[25]	ChCl	HFIP		TAPB	DHA	COF-DES	solvent
[26]	ChCl	OA		Tp	Pa	TpPa	solvent
[28]	ChCl	urea		Tp	Pa, TAPB	TpPa, Tp-TAPB	solvent, catalyzator

ChCl: choline chloride; 2-Mim: 2-methylimidazole; e-urea: ethylene urea; TBAB: tetrabutylammonium bromide; Gly: glycerol; EG: ethylene glycol; Im: imidazole; HFIP: hexafluoroisopropanol; OA: oxalic acid dihydrate; DHTA: 2,5-dihydroxyterephthalic acid; PTA: phthalic acid; SDS: sodium dodecyl sulfate; Tp: 1,3,5-triformylphloroglucinol; TFPM: tetrakis(4-formylphenyl)-methane; TFPT: 2,4,6-tris(5-formyl-2-pyridinoxy)-1,3,5-triazine; TAPB: 1,3,5-tris(4-aminophenyl) benzene; Pa: *p*-phenylenediamine; Ha: hydrazine; Azo: 4,4'-azodianiline; Bpy: 2,2'-bipyridine-4,4'-diamine; DHA: 2,5-dihydroxyterephthalaldehyde; SDA: structure-directing agent.

## 2 POFs在固相萃取中的应用

在实际样品分析中,由于样品的组成复杂,且目标分析物的含量通常处于痕量或超痕量水平,很难直接用仪器进行检测。因此,在分析检测前对样品进行分离与富集是十分必要的。固相萃取具有简单、高效、易自动化和有机溶剂消耗少等优点,已被广泛应用于分析检测工作中。根据萃取材料的形式,固相萃取可以分为柱固相萃取(column solid-phase extraction, CSPE)、固相微萃取(solid-phase microextraction, SPME)、磁性固相萃取(magnetic solid-phase extraction, MSPE)、分散固相萃取(dispersive solid-phase extraction, dSPE)和搅拌棒吸附萃取法(stir bar sorptive extraction, SBSE)等。以下就基于DES合成的MOFs和COFs所涉及的固相萃取进行介绍(表2)。

**表 2:** 基于DES的MOFs和COFs在固相萃取中的应用

Ref.	Sorbent	Type of SPE	Targets	Samples
[25]	TAPB-DHA	CSPE	flavanoid	hemb bean powder, medicus flowers
[29]	MIL-53(Al)	CSPE	phthalate	none
[30]	MIL(Al)-53-DES@MIPs	CSPE	aflatoxin	wheat, rice, corn
[31]	MIL-101(Cr)	CSPE	phenols	lake water
[32]	MIL-101(Cr)@DES	CSPE	imidacloprid	tea, water
[33]	DES@UMCM-1MOF/MIP	SPME	antibiotic	meat, dairy products
[34]	UMCM-1-DES/MIPs	SPME	phthalate	yogurt, water, cooking oil
[35]	Fe_3_O_4_@ZIF-8@Polymer	MSPE	organophosphorus pesticide	river water, pear, cabbage
[36]	ILM/ZIF-8	MSPE	aflaoxin	milk
[37]	Fe_3_O_4_/ZIF-8/IL	MSPE	pyrethroid pesticides	tea infusion
[38]	MM/ZIF-8@DES_5_	MSPE	pyrethroid pesticides	tea drink
[39]	M-ZIF-8@DES	MSPE	pyrethroid pesticides	environmental water sample
[40]	polydimethylsiloxane/MIL-100(Fe)	SBSE	triazines	environmental water sample
[41]	DES-Fe_3_O_4_@MIL-100(Fe)	dSPE	dopamine, adrenaline, and norepinephrine	serum, urine
[42]	Fe_3_O_4_@TAPB-DHA	MSPE	polycyclic aromatic hydrocarbons	sausage, roast duck meat
[43]	TAPB-DHA	SPME	flavanoid	biological sample

MIP: molecularly imprinted polymer; IL: ionic liquid; CSPE: column solid-phase extraction; MSPE: magnetic solid-phase extraction; dSPE: dispersive solid-phase extraction; SBSE: stir bar sorptive extraction.

### 2.1 MOFs在固相萃取中的应用

#### 2.1.1 柱固相萃取

柱固相萃取是一种常用的样品预处理技术,用于从复杂的样品中分离和富集目标分析物。它在分析化学、环境监测、食品检测、药物分析等领域得到了广泛应用。柱固相萃取的基本原理是利用固相材料的吸附特性将目标分析物从样品基质中分离出来。柱固相萃取是最传统的固相萃取方法,主要包括柱预处理、样品加入、柱洗涤和洗脱步骤。2016年,Shu等^[29]^首次利用MIL-53(Al)作为液相色谱分析的固定相,用于磷酸二甲酯的分离。发现当以MeOH-H_2_O(92∶8, v/v)作为流动相时,5种邻苯二甲酸酯(邻苯二甲酸丁酯、邻苯二甲酸二丁酯、邻苯二甲酸二乙酯、邻苯二甲酸二乙酯和邻苯二甲酸二甲酯)在12 min内实现了基线分离,且具有可靠的重现性。但是,MIL-53(Al)粒度分布宽且不规则,填充柱效率低且背压高。因此,需要提高MOF的粒径均匀性和适当的形态。2023年,Kardani等^[30]^采用由ChCl和Gly组成的DES改性MIL-53(Al),并基于合成的MIL-53(Al)-DES与分子印迹聚合物(molecularly imprinted polymers, MIPs)合成了MIL-53(Al)-DES@MIPs免疫亲和柱,用于提取和预浓缩黄曲霉毒素AFB1、AFB2、AFG1和AFG2。在优化条件下,4种黄曲霉毒素的检出限(LOD)范围为0.023~0.033 μg/kg(*S/N*=5),在0.1~400 μg/kg的范围内,标准曲线呈线性关系,相关系数(*R*^2^)>0.9970。对于3种不同浓度水平的黄曲霉毒素,单柱的日内和日间相对标准偏差(RSD)为1.3%~4.4%,柱间RSD为4.3%~6.7%,DES的使用提高了MOF颗粒的稳定性。该萃取柱具有灵敏度高、选择性好、定量提取效率高、线性范围宽、重复性好等优点。

Yang等^[31]^通过制备MIL-101(Cr)掺杂聚合物整体柱,采用在线SPE-HPLC方法检测水中酚类化合物。该方法具有线性好、精密度好、检出限低等优点,在实际应用中具有很大的可行性,其中MIL-101(Cr)对提高酚类化合物的萃取效率起着关键作用。为了增强MIL-101(Cr)对分析物更好的吸附和分离性能,以及提高回收率和重复使用性能,可以尝试通过DES进行改性。Ozalp等^[32]^利用由ChCl和尿素组成的DES对MIL-101(Cr)进行改性,制备出MIL-101(Cr)@DES纳米复合材料,并将其用作吸附剂,用于固相萃取药茶注射液和水样中的吡虫啉,并进行HPLC分析。该方法表现出良好的灵敏度、低吸附剂用量、短吸附时间、高回收率和可重复利用性。固相萃取过程中使用的吸附剂仅为10 mg,吸附时间为3 min,富集倍数为20倍,可连续重复使用15次。通过DES改性,MIL-101(Cr)的比表面积增加了25倍,孔隙结构增强,吸附累积体积增大,从而使MIL-101(Cr)的吸附性能得到显著提升。

#### 2.1.2 固相微萃取

固相微萃取是柱固相萃取的一种小型化形式,能够将采样、预浓缩和萃取步骤整合为一个步骤,具有低成本操作中极低或无需溶剂消耗等优点。然而,MOF作为吸附剂在固相微萃取方法中的应用存在化学稳定性差的问题。因此,采用DES对MOF材料进行改性是一种可行的方法,可提高吸附剂的化学稳定性。2020年,Mirzajani等^[33]^首次将UMCM-1 MOF应用于固相微萃取中,并通过由ChCl和Gly组成的DES对其进行改性,在绿色化学的条件下实现UMCM-1化学稳定性的提高。该研究团队基于UMCM-1 MOF-DES/MIPs制备了中空纤维和单片纤维,并采用中空纤维液膜保护固相微萃取法(HFLMP-SPME)对酸奶、水和食用油中邻苯二甲酸酯(PEA)进行了GC-FID检测。该方法的LOD(*S/N*=3)为0.008~0.03 μg/L, LOQ(*S/N*=10)为0.028~0.12 μg/L,日内和日间RSD分别为2.4%~4.7%和2.6%~3.4%。与传统的金属有机框架/分子印迹聚合物相比,MOF-DES/MIPs单片纤维具有更大的吸附容量,以及更快的吸附和解吸动力学,从而提高了分析物在聚合物表面的吸附和定向目标,增强了目标化合物在聚合物结构中的结合率。

Kardani等^[34]^基于由ChCl和Gly组成的DES,结合UMCM-1和MIP制备了单片纤维(DES@UMCM-1-MOF/MIP)。随后,将5个单片纤维黏结在一起,得到多个单片纤维(MMF),并利用SPME-HPLC-UV提取了39种不同类别的抗生素。总抗生素在5.0~1400 μg/L范围内呈线性关系,*R*^2^≥0.995,LOD和LOQ分别为1.1~2.3 μg/L和3.3~7.6 μg/L,不同样品的平均加样回收率为95.1%~100.0%。该方法具有非常高的选择性和纤维吸附容量,无需添加进一步的纯化程序来去除共萃取剂,并且在应用于复杂样品中抗生素测定方面具有潜力。

#### 2.1.3 磁性固相萃取

在磁性固相萃取过程中,磁性吸附剂直接分散于样品溶液中,高效地萃取分析物,然后通过外加磁场的作用快速分离。相对于传统的填充式固相萃取,磁性固相萃取可以避免与吸附剂填料相关的典型问题,如高压和填料床堵塞,从而具有操作简便、省时省力以及提取效率高等优点。在磁性固相萃取中,磁性吸附剂直接影响萃取效率,也决定了方法的灵敏度和选择性,因此在这一过程中扮演着至关重要的角色。Wan等^[35]^利用原位自组装和沉淀聚合的方法成功制备了核-壳-壳磁性纳米球Fe_3_O_4_@ZIF-8@聚合物,并将其应用于磁性固相萃取材料,从河水、梨和白菜中提取9种有机磷农药(OPPs)。该方法在0.2~200 μg/L范围内具有良好的线性,*R*^2^≥0.9991。在*S/N*=3时,河水中OPPs的LOD和LOQ分别为0.0002~0.005 μg/L和0.0007~0.017 μg/L,梨和白菜的LOD和LOQ分别为0.006~0.185 μg/kg和0.023~0.618 μg/kg,这些值远低于中国和欧盟对OPPs的最大残留限量,方法的日内和日间RSD分别为1.2%~7.3%和5.4%~9.7%。这些结果显示出Fe_3_O_4_@ZIF-8@聚合物具备出色的磁分离性能和多目标吸附能力。另一方面,Gao等^[36]^基于IL、Fe_3_O_4_和ZIF-8制备了ILM/ZIF-8,并将其用于检测牛奶样品中的黄曲霉毒素。方法的LOD(*S/N*=3)和LOQ(*S/N*=10)分别为2.3~8.1 ng/L和7.5~26.7 ng/L,日内和日间RSD分别为3.3%~6.3%和4.2%~7.2%。此外,Huang等^[37]^也制备了Fe_3_O_4_/ZIF-8/IL磁性吸附剂,用于茶水中4种拟除虫菊酯的分析。联苯菊酯在0.5~50 mg/L范围内,氯菊酯、氯氰菊酯和氟氰菊酯在0.5~500 mg/L范围内,表现出良好的线性关系。当*S/N*=3时,LOD为0.0065~0.1017 μg/L。这项研究表明,磁性ZIF-8与IL结合可以提高ZIF-8对拟除虫菊酯的吸附能力,同时提升IL在磁性固相萃取中的适用性。

受IL的启发,Huang等^[38]^继续采用具有类似性质的绿色溶剂DES对ZIF-8进行改性。他们利用由四丁基氯化铵和十二醇组成的DES,结合多壁碳纳米管修饰的ZIF-8(MWCNTs-ZIF-8),成功制备了一种新型复合材料MM/ZIF-8@DES,并将其用作茶饮料中6种拟除虫菊酯的磁性固相萃取吸附剂。通过分析含有不同质量浓度(0.5、1、2、5、10、20、50、100、200和500 μg/L)6种拟除虫菊酯的样品溶液,证明该方法具有良好的线性关系,*R*^2^为0.9905~0.9925。*S/N*=3时,LOD为0.08~0.33 μg/L,日内和日间RSD分别小于5.58%和8.58%。这表明该方法可以有效检测茶饮料中拟除虫菊酯类农药残留。与此同时,与M-MWCNTs和MM/ZIF-8相比,MM/ZIF-8@DES表现出最佳的拟除虫菊酯吸附性能,而MM/ZIF-8次之。这可以部分归因于MM/ZIF-8@DES具有更大的表面积,从而增强了其吸附能力。此外,MM/ZIF-8@DES中DES的疏水性和可能的HBA和HBD性质,也在一定程度上提高了其吸附性能。

Liu等^[39]^基于1-甲基-3-辛基咪唑氯和十一醇构成的DES对磁性ZIF-8进行了改性,制备了复合材料M-ZIF-8@DES,并将其应用于环境水样中拟除虫菊酯类农药的磁性吸附。他们通过气相色谱-三重四极杆质谱进行了氰戊菊酯、氟氯氰菊酯、氯氰菊酯和氟氰菊酯4种拟除虫菊酯的测定(如图3所示)。M-ZIF-8@DES具有典型的超顺磁性、大比表面积和孔隙体积,有助于实现环境水样中拟除虫菊酯类化合物的快速富集。在1~500 μg/L范围内,该方法展现出良好的线性关系(*R*^2^≥0.9916)。*S/N*=3时,LOD为0.05~0.21 μg/L,日内和日间RSD均小于9.40%。这些结果显示出此方法不仅具备良好的线性特性和低检出限,还具有较高的测量精确度,为基于DES改性ZIF-8去除环境水样中农药的方法奠定了基础。

**图 3:**
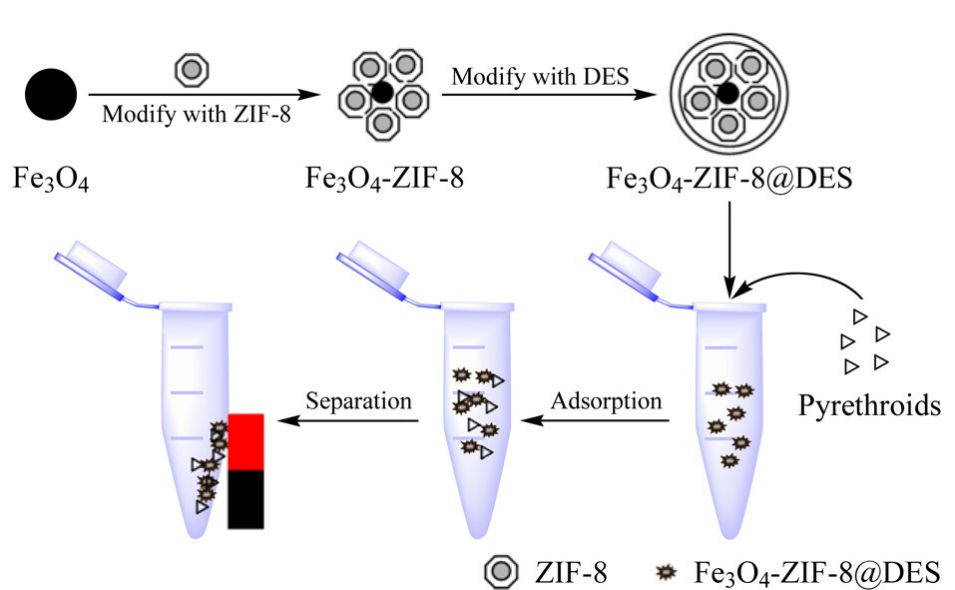
M-ZIF-8@DES的制备示意图以及磁性固相萃取方法的步骤^[39]^

#### 2.1.4 分散固相萃取

分散固相萃取是将微米或纳米级吸附剂分散到样品溶液中,从而促进吸附剂与目标物之间的强相互作用。目标分析物在溶剂中被吸附、富集,之后通过少量的解吸溶剂进行洗脱,从而获得目标分析物。2017年,Lei等^[40]^基于MIL-100(Fe),通过溶胶凝胶技术制备了聚二甲基硅氧烷(PDMS)/MIL-100(Fe)涂层搅拌棒,并将其用于吸附萃取,建立了PDMS/MIL-100(Fe)-SBSE-超声波辅助液体解吸-HPLC-UV测定环境水样中三嗪类化合物的方法。该涂层搅拌棒的制备具有良好的重现性,并且与其他商用涂层搅拌棒相比,对目标三嗪类化合物表现出更高的萃取效率。为了进一步提升MIL-100(Fe)的萃取效率,可以通过DES进行改性,从而使其具备更小的空腔尺寸和更多的开放金属位点。

在2023年,Khezeli等^[41]^利用ChCl和Urea/EG构成的DES,对接枝了邻苯二酚的Fe_3_O_4_@MIL-100 (Fe)核壳纳米颗粒进行改性。他们应用这一改性后的磁性吸附剂进行分散微固相萃取,以提取生物样品中的多巴胺、肾上腺素和去甲肾上腺素,然后通过HPLC-UV进行检测。萃取过程包括将含分析物和吸附剂的样品水溶液进行超声处理,随后使用涡流搅拌器进行5 min的搅拌,然后通过外置磁铁收集磁性吸附剂,除去水溶液,加入50 μL洗脱溶剂磷酸盐缓冲液-DES(7∶3, v/v),随后进行超声处理,再用外置磁铁收集磁性颗粒,用微型注射器抽出洗脱溶剂,最终注入高效液相色谱系统进行分析。该方法在优化条件下,多巴胺、肾上腺素和去甲肾上腺素在1~300 μg/L范围内展现出良好的线性关系,*R*^2^>0.9966。LOD和LOQ分别为0.22~0.36 μg/L和0.78~1.20 μg/L,平均回收率为91.4%~103.4%。该方法已成功应用于加标人尿和血清样品中目标分析物的测定。DES的引入显著提高了Fe_3_O_4_@MIL-100 (Fe)的萃取效率。

### 2.2 COFs在固相萃取中的应用

Chen等^[42]^利用磁性Fe_3_O_4_纳米粒子为核心,以TAPB和DHA为单体,构建了一种新型磁性核-壳结构(Fe_3_O_4_@TPB-DHA)纳米复合材料。基于这一材料,结合高效液相色谱-二极管阵列检测,建立了一种简便快速的方法,用于磁性固相萃取食品样品中11种多环芳烃(PAHs)。在不同含量(2、2.5、5、10、25、50、100、200 μg/kg)的香肠提取物中,该方法表现出良好的线性关系,*R*^2^为0.9954~0.9988。方法的LOD为1.84~8.35 μg/kg, LOQ为6.12~27.47 μg/kg。方法的日内和日间RSD均小于5.8%。这表明该方法能够有效富集食品样品中的PAHs,并且能够检测出其中微量的PAHs。此外,该方法还具有在提取其他芳香化合物方面的潜在应用前景。

Gao等^[25]^以TAPB和DHA为单体,基于ChCl和HFIP组成的DES合成了具有高结晶度的COF-DES,并将其用于富集天然药物马齿苋花中的黄酮类化合物。COF-DES的比表面积为444.56 m^2^/g,远高于Fe_3_O_4_@COF的122.43 m^2^/g,初步显示了COF-DES良好的吸附能力。分析质量浓度为0.1~100 μg/mL的3种黄酮类化合物(杨梅素、槲皮素、芦丁)的样品溶液,发现该方法具有良好的线性关系(*R*^2^>0.9922), LOD和LOQ分别为0.1~18 ng/mL和0.5~30 ng/mL,日间和日内RSD分别为0.64%~1.93%和1.01%~6.86%,回收率为77.87%~107.88%。与通常用于黄酮类化合物分离富集的大孔树脂(如AB-8、D101)相比,少量的COF-DES在吸附效果上超越了10倍以上的大孔树脂,基于COF-DES的固相萃取柱对黄酮类化合物表现出更佳的吸附性能。

在此基础上,Quan等^[43]^将COF-DES用作固相微萃取纤维的涂层,用于提取和检测生物样品中的黄酮类苷元或其代谢物。基于COF-DES的固相微萃取纤维具有合适的孔径和丰富的官能团,具有出色的大分子排阻能力,对木犀草素和槲皮素具有较高的吸附能力和良好的选择性。木犀草素和槲皮素在该方法中的线性范围分别为0.1~150 μg/mL和0.025~1 μg/mL(*R*^2^=0.9988),有低的LOD(10~50 ng/mL)和LOQ(25~100 ng/mL)。与传统的蛋白沉淀法相比,基于固相微萃取纤维的萃取方法具有更好的萃取效率,这表明该方法在生物样品中富集黄酮类苷元或其代谢物方面具有广阔的应用前景。

## 3 总结与展望

总之,采用DES合成MOFs和COFs已成为一种高效绿色的策略。当DES被用作合成过程的溶剂时,合成方法比传统方法更环保、无毒,且所获得的MOFs和COFs通常具有更高的结晶度、更大的比表面积以及更好的水热稳定性。甚至有时可在DES中合成一些在传统溶剂中难以获得的COFs。当DES作为结构导向剂时,其成分参与MOFs和COFs的形成,影响其结构和性能,从而实现特定的结构设计。

鉴于MOFs和COFs具备较大的比表面积、丰富的活性位点以及高度稳定性等优势,它们在固相萃取领域具有广泛的应用前景。然而,基于DES合成MOFs和COFs的研究仍在不断发展中,将其应用于固相萃取领域的工作尚属少见,而更多性能优越的MOFs和COFs仍有待挖掘。未来,应持续推动基于环境友好的绿色溶剂DES的合成方法,用以制备MOFs、COFs甚至其他种类的POFs,并考虑将在高富集效率方面表现出色的POFs运用于固相萃取领域,并最终将其商业化。
